# Identification and targeting of selective vulnerability rendered by tamoxifen resistance

**DOI:** 10.1186/s13058-020-01315-5

**Published:** 2020-07-29

**Authors:** Madhurendra Singh, Xiaolei Zhou, Xinsong Chen, Gema Sanz Santos, Sylvain Peuget, Qing Cheng, Ali Rihani, Elias S. J. Arnér, Johan Hartman, Galina Selivanova

**Affiliations:** 1 Department of Microbiology, Tumor and Cell Biology, Karolinska Institutet, SE-171 65 Stockholm, Sweden; 2 Department of Oncology and Pathology, Karolinska Institutet, CCK, 171 76 Stockholm, Sweden; 3 Division of Biochemistry, Department of Medical Biochemistry and Biophysics, Karolinska Institutet, SE-171 65 Stockholm, Sweden

**Keywords:** Tamoxifen resistance, SULT1A1, RITA, Aminoflavone, Oncrasin-1, TrxR1

## Abstract

**Background:**

The estrogen receptor (ER)-positive breast cancer represents over 80% of all breast cancer cases. Even though adjuvant hormone therapy with tamoxifen (TMX) is saving lives of patients with ER-positive breast cancer, the acquired resistance to TMX anti-estrogen therapy is the main hurdle for successful TMX therapy. Here we address the mechanism for TMX resistance and explore the ways to eradicate TMX-resistant breast cancer in both in vitro and ex vivo experiments.

**Experimental design:**

To identify compounds able to overcome TMX resistance, we used short-term and long-term viability assays in cancer cells in vitro and in patient samples in 3D ex vivo, analysis of gene expression profiles and cell line pharmacology database, shRNA screen, CRISPR-Cas9 genome editing, real-time PCR, immunofluorescent analysis, western blot, measurement of oxidative stress using flow cytometry, and thioredoxin reductase 1 enzymatic activity.

**Results:**

Here, for the first time, we provide an ample evidence that a high level of the detoxifying enzyme SULT1A1 confers resistance to TMX therapy in both in vitro and ex vivo models and correlates with TMX resistance in metastatic samples in relapsed patients. Based on the data from different approaches, we identified three anticancer compounds, RITA (*R*eactivation of p53 and *I*nduction of *T*umor cell *A*poptosis), aminoflavone (AF), and oncrasin-1 (ONC-1), whose tumor cell inhibition activity is dependent on SULT1A1. We discovered thioredoxin reductase 1 (TrxR1, encoded by *TXNRD1*) as a target of bio-activated RITA, AF, and ONC-1. SULT1A1 depletion prevented the inhibition of TrxR1, induction of oxidative stress, DNA damage signaling, and apoptosis triggered by the compounds. Notably, RITA efficiently suppressed TMX-unresponsive patient-derived breast cancer cells ex vivo.

**Conclusion:**

We have identified a mechanism of resistance to TMX via hyperactivated SULT1A1, which renders selective vulnerability to anticancer compounds RITA, AF, and ONC-1, and provide a rationale for a new combination therapy to overcome TMX resistance in breast cancer patients. Our novel findings may provide a strategy to circumvent TMX resistance and suggest that this approach could be developed further for the benefit of relapsed breast cancer patients.

## Background

Despite the remarkable clinical success of TMX therapy, patients with metastatic disease develop therapeutic resistance after receiving the hormonal therapy due to various mechanisms, including amplification of Her2, increased Mitogen-activated protein kinases (MAPK) signaling, alterations of ER or cytochrome (CYP) gene expression [1–3]. Notably, about 30–40% of patients who receive adjuvant TMX ultimately recur and die from their disease, presenting a huge clinical challenge [4, 5]. Therefore, identification of drug combinations for therapy of non-responder patient’s group is of utmost importance.

TMX is a prodrug metabolized by products of CYP genes to a more active metabolite 4-hydroxy-tamoxifen (4OH-TMX) with high binding affinity to ER [6]. Patients with CYP activity compromised either by specific single nucleotide polymorphisms (SNPs) of CYP2D6 or mutation in CYP genes have worst outcome after TMX treatment [7], suggesting that detoxification enzymes could be potential biomarkers. Another detoxification enzyme Sulfotransferase 1A1 (SULT1A1) is a member of the sulfotransferase enzymes which can eliminate 4OH-TMX [8]. It was shown that patients with low activity of SULT1A1 due to SNP in the *SULT1A1* gene and who received adjuvant TMX or chemotherapy display better survival [9]. In contrast, other studies suggest that SNP conferring normal SULT1A1 activity is associated with better survival upon TMX [10, 11]. Therefore, it appears important to resolve this controversy and to establish an association between SULT1A1 and TMX.

In this study, we have identified SULT1A1 to be upregulated in relapsed metastatic breast tumors in patients who received TMX therapy. We reasoned that SULT1A1-dependent drugs (or their metabolites) might overcome resistance to TMX. We found that the tumor suppressor effect of three anticancer compounds, RITA [12–14], aminoflavone (AF; (5-amino-2-(4-amino-3-fluorophenyl)-6,8-difluoro-7-methylchromen-4-one; NSC 686288) [15], and derivative of oncrasin-1 (ONC-1; (1- {(4-chlorophenyl)methyl}-1H-indole-3-carboxaldehyde) [16], is dependent on the expression of SULT1A1, in line with previous reports [17–19]. Recently, we have identified cancer cell-specific oxidative-dependent inhibition of the transcription of several oncogenes by RITA, AF, and ONC-1 [20]. Moreover, we identified a common target for these compounds, TrxR1, and demonstrated that targeting TrxR1 by the three compounds is SULT1A1-dependent. We found that RITA and AF can overcome TMX resistance. Our findings can open the way to new treatment modalities for relapsed breast cancer patients.

## Methods

### Cell lines

MCF7 (ATCC), MCF7 TMX^R^ spontaneously obtained in our lab and tamoxifen-resistant MCF7/LCC2 (kindly provided by Nils Brünner, University of Copenhagen) were cultured in phenol-red-free DMEM supplemented with 10% FBS (Hyclone), 2 mM l-glutamine, 100 U/ml of penicillin, and 100 mg/ml of streptomycin (Sigma-Aldrich). The TMX-resistant MCF7/LCC2 cells were selected stepwise against increasing concentrations of 4-OH-TMX. Selection began with 1 nM and increased by half a decade after three consecutive passages and the final concentration used was 1 μM 4-OH-TMX), and maintained in 1 μM 4-OH-TMX [21]. HCT116 (ATCC), A375 (ATCC), H1299 (ATCC), GP5d (ATCC), A431 (ATCC), and MDAMB-231 (ATCC) were grown in DMEM supplemented with 10% FBS, and antibiotics. Primary patient-derived KADA line (kindly provided by Rolf Kiessling, Karolinska hospital) was cultured in IMDM. SJSA-1 (ATCC), U2OS (ATCC), and SKMEL28 (kindly provided by Lars-Gunnar Larsson, Karolinska Institutet) were cultured in RPMI 1640 with 10% FBS and antibiotics. The pretreatment (96 h) of 50 nM sodium selenite (Sigma-Aldrich) was performed in the cell lines only when TrxR1 activity measurement was performed. CRISPR/Cas9-mediated SULT1A1 deletion was performed in stable Cas9-expressing MCF7 and HCT116 cells using gRNAs targeting exon 4 - ATCTGGGCCTTGCCCGACGA and exon 7 - AATTGAGGGCCCGGGACGGT. Cas9 expressing plasmid was provided by Vera Grinkevich, Welcome Trust Sanger Institute, Cambridge, UK.

A375 and SJSA-1 cells, stably expressing SULT1A1 cDNA (OriGene, #RC201601L1), were generated by lentivirus transduction using standard procedure [22].

### Clinical material

Between November 2017 and May 2018, fresh breast cancer specimens from 11 patients were collected at the Karolinska University Hospital and Stockholm South General Hospital. Experimental procedures and protocols were approved by the regional ethics review board (Etikprövningsnämnden) in Stockholm, Sweden, with reference numbers 2016/957-31 and 2017/742-32. The material was obtained according to Stockholm Medical Biobank approval number Bbk1730.

### Compounds

RITA (NSC652287) and aminoflavone (NSC686288) were obtained from the National Cancer Institute (NCI), oncrasin-1 was from Santa Cruz Biotechnology, and 4OH-TMX and resveratrol were purchased from Sigma-Aldrich. We have tested different concentrations of 4OH-TMX (from 10 nM to 1 μM) in ex vivo samples and from 100 nM to 6 μM range of concentrations in MCF7 cells in a short-term viability experiment. The concentration of 4OH-TMX which we used is consistent with several reports in which 4OH-TMX was used in a short-term experiment [23–25]. The TMX-resistant MCF7-LCC2 cells were treated with ≥1 μM 4OH-TMX. The compound concentrations and durations of treatment are mentioned in the figure legends.

### 3D ex vivo model

Our 3D ex vivo model is based on the study of Vaira et al. [26], in which they established an organotypic culture model that maintains original tumor microenvironment in the presence of 20% inactivated FBS. We further modified this protocol by collecting the breast cancer clinical samples with superficial scraping, instead of tumor tissue section, which allows us to culture all the components from parental tumors maintaining tumor heterogeneity and epithelial-stromal interactions [27].

Primary cancer cells were collected by superficial scrapings from surgically resected breast tumors [27]. The cell smears were immediately processed by lysis of red blood cells, followed by trypsinization (Thermo Fisher Scientific, MA, USA) and filtration (Miltenyi Biotec, Bergisch Gladbach, Germany) into single-cell suspensions and three time washing with PBS. The last cell pellet was re-suspended with selective DMEM F/12 medium supplied with 20% FBS and Antibiotic-Antimycotic (all from Thermo Fisher Scientific, MA, USA), then seeded at the density of 1500 cells in 60 μL medium per well into 96-well plate (Sigma Aldrich, MO, USA) using the MultiDrop Combi dispenser (Thermo Fisher Scientific, MA, USA). Cells were then divided into either vehicle group or tamoxifen treatment group, where DMSO or 1 μM of 4OH-TMX- (both from Sigma Aldrich, MO, USA) were supplied to the culture and replenished every 48 h. RITA or equivalent volume of DMSO was added to both experiment groups at day 6. The cell viability was assessed using CellTiter-Glo 3D assay (Promega, WI, USA) according to the manufacturer’s instruction, and reading luminescence by a Tecan spark 10M microplate reader (Tecan, Männedorf, Switzerland) at day 9 as the experiment end point.

### Cell viability and growth suppression assays

For colony formation assay, 25,000 MCF7 cells were seeded in a 12-well format, pretreated with 4OH-TMX for 6 days. After pretreatment, the cells were co-treated with indicated low dose of RITA, AF, and ONC-1 for another 6 days and stained with crystal violet (CV). For HCT116 cells, 100,000 cells were seeded in 12-well plates, treated with indicated concentration of drugs for 3 days and followed by CV staining. For short-term viability assay, 3000 cells/well were plated in a 96-well plate and treated with indicated concentration of drugs for 72 h, and cell viability was assessed using resazurin assay according to the manufacturer’s instructions. No additional selenium was added to culture medium in different cellular assays.

### Immunofluorescence staining

Primary breast cancer cells were cultured in low-adherent plate in the presence or absence of 1 μM 4OH-TMX. 4OH-TMX was replenished every 48 h for 6 days, and cytospin was performed at speed of 50×*g* for 10 min. Cells were then fixed at − 20 °C with ice-cold 1:1 mixed methanol and acetone (Sigma-Aldrich, MO, USA) for 20 min. Subsequently, cells were incubated in IF buffer (4% bovine serum albumin, 0.05% saponin in PBS) for 1 h and stained in IF buffer with the primary antibodies at 1:200 dilution: α-SULT1A1 ab191069 (green staining) or ab124011 (red staining), both from Abcam. DNA was detected using 4,6-diamidino-2-phenylindole (DAPI, 1 mg/ml: Sigma). Analyses were performed with an automated Olympus IX73 inverted microscope. Quantitative immunofluorescence analysis was performed using Fiji/ImageJ software (https://imagej.net/Fiji).

### Real-time quantitative PCR assay

The total RNA was extracted using Aurum™ Total RNA Mini Kit (Bio-Rad), according to the manufacturer’s protocol, followed by amplification and cDNA synthesis using MessageBOOSTER™ cDNA Synthesis Kit (Lucigen) or iScript cDNA synthesis kit (Bio-Rad) for qPCR, as described by the manufacturer. Real-time PCR was conducted with SsoAdvanced™ Universal SYBR® Green Supermix (Bio-Rad). GAPDH, RPL13A, and RPLP0 were used as housekeeping genes. The SULT1A1 primer sequences are FP- CGGCACTACCTGGGTAAGC and RP- CACCCGCATGAAGATGGGAG.

### Correlation sensitivity analysis

NCI-60 Analysis Tools provided by CellMiner [28] (https://discover.nci.nih.gov/cellminer/) and the NCI-60 cell line panel with associated drug screens were used to calculate the drug sensitivity Pearson correlation coefficient between SULT1A1 mRNA expression and RITA, oncrasin-1, and aminoflavone. The 50% growth inhibitory concentration (GI_50_) values of compounds were used for calculations.

### shRNA screen

Genome-wide pooled short hairpin RNA (shRNA) screen to identify genes which confer resistance to RITA was performed in MCF-7 cells. Briefly, cells infected with lentivirus library of 27,290 shRNAs targeting 5046 human genes were selected on Puromycin, allowed to propagate for 1 week before treatment with 1 μM RITA. Sequencing of the barcodes from survived cells gave 20 million individual scores of shRNAs. To identify shRNAs whose abundance was significantly different between control (DMSO) and RITA-treated cells, we used the following criteria: (i) *P* value < 0.1, (ii) FDR < 0.3, and (iii) *P* values of the weighted *Z*-scores, *P* (w*Z*) < 0.1, which integrate the information from multiple shRNAs targeting a single gene, thus minimizing the impact of possible off-target effects.

These procedures allowed us to identify shRNAs which confer resistance to RITA. The detailed experimental procedure and the data processing are described in [29].

### RNA-seq analysis

SULT family gene expression was analyzed using RNA-seq data obtained in primary breast cancer and liver metastatic tumors from three patients treated with endocrine therapy for 5 years [30]. Read count data was downloaded using the accession number GSE58708 and normalized with DESeq2 [31] for clustering. Hierarchical clustering was computed and visualized using GENE-E software (https://software.broadinstitute.org/GENE-E/index.html).

### Oxidative stress measurement

Cells were treated as mentioned in figure legends and incubated 30 min with 10 μM DCF-DA in serum-free medium. Afterward, cells were trypsinized and washed with PBS, and fluorescence was analyzed by a FACSCalibur flow cytometer (BD Biosciences) using FL1-H channel.

### TrxR1 enzymatic activity measurement

Total cell lysates (40 μg protein) from cells treated with RITA, AF, and ONC-1 were subjected to TrxR-dependent Trx-coupled insulin disulfide reduction assay as described before [32].

### Western blot analysis

The extraction of total cell lysates and western blot were performed according to standard procedure. The antibodies used for immunoblotting were as follows: SULT1A1 (ab124011 and ab191069, Abcam), p53 (sc-126, Santa Cruz Biotechnology), PARP (#95423, Cell Signaling), and γH2AX (#07-164, Millipore). Anti-β-actin monoclonal antibody (Millipore) was used as loading control. The horseradish peroxidase-coupled secondary antibodies (Jackson ImmunoResearch) and SuperSignal™ West Dura Extended Duration Substrate detection system (Thermo Fisher Scientific), and images were visualized using ChemiDoc Imaging System (Bio-Rad).

### Statistical analysis

For all experiments, statistical analyses were performed using GraphPad Prism 5. Results are given as mean ± s.d. To evaluate statistical significance, Student’s *t* test (unpaired) or one-way ANOVA with Bonferroni’s multiple comparison test were performed and mentioned in the figure legends. *P* values ≤0.05 were considered statistically significant.

## Results

### SULT1A1 gene upregulation upon TMX treatment

**Fig. 1:**
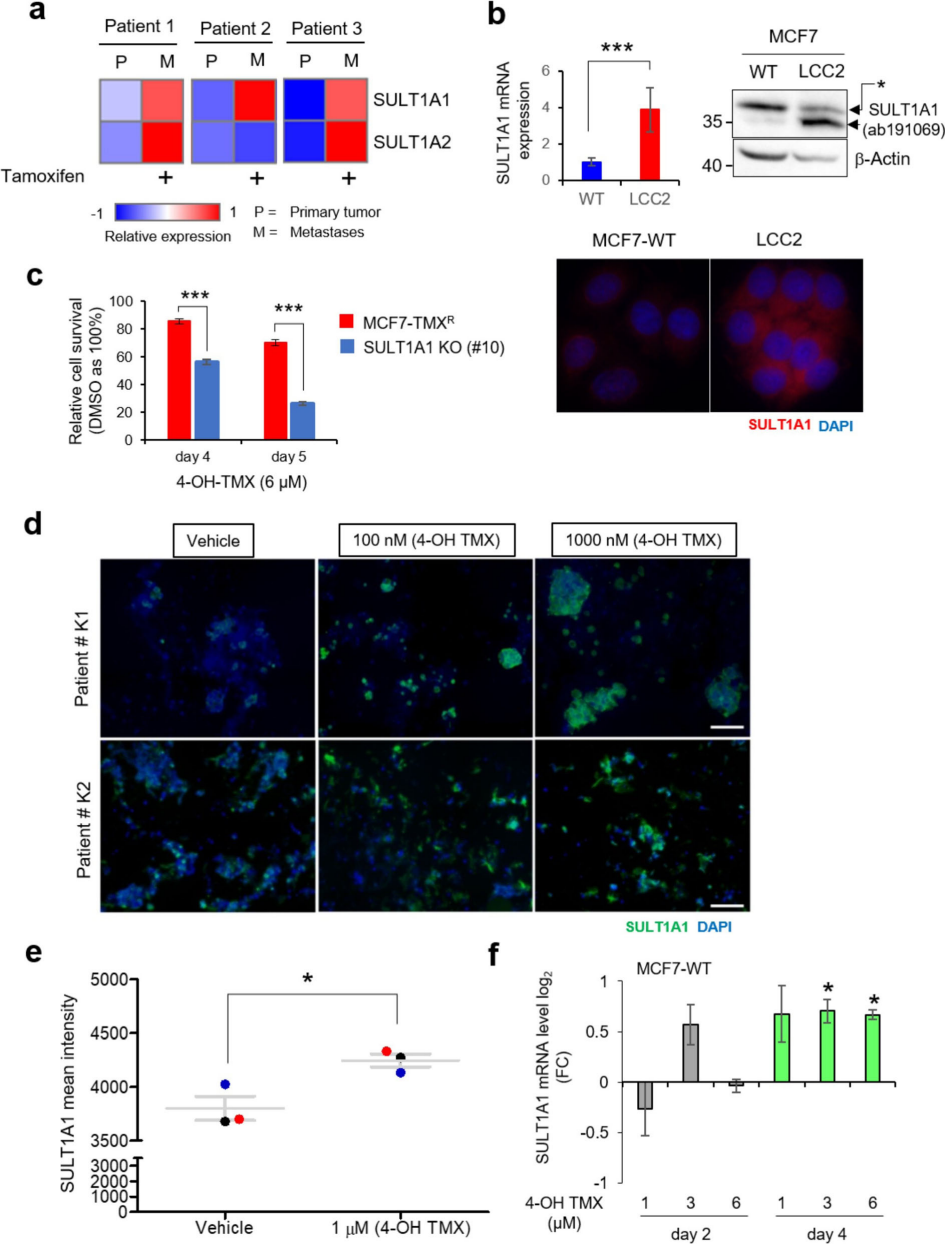
TMX resistance in breast cancer patient samples and cell lines is associated with elevated SULT1A1 expression. **a** Heatmap of SULT1A1 and SULT1A2 genes differentially expressed in matched primary tumor and liver metastasis samples from breast cancer patients as assessed by RNA-seq. SULT1A1 expression is stronger in the liver metastasis, sampled after TMX treatment. **b** SULT1A1 mRNA expression and protein level were analyzed in MCF7-WT and its TMX-resistant (TMX^R^) derivative LCC2 cells, using qRT-PCR (***p < 0.001, Student’s *t* test) (left panel), WB (right panel), and immunofluorescence (low panel). The asterisk indicates non-specific band detected by SULT1A1 (ab191069) antibody. **c** SULT1A1 deletion (blue bars, #10) in spontaneous TMX^R^ clone of MCF7 cells (red bars) confers sensitivity to 6 μM 4OH-TMX, as determined by resazurin assay (***p < 0.001, one-way ANOVA with Bonferroni’s multiple comparison test). **d** Immunofluorescence analysis of SULT1A1 protein (in green, using ab191069 antibody) after 6 days of 4OH-TMX treatment of breast cancer patient cells cultured in 3D ex vivo. **e** ImageJ quantification of total mean SULT1A1 intensity in samples treated as in **d**. Black, red, or blue dot represent patients K1, K2, and K3, respectively, (Table S1) (*p < 0.05, Student’s *t* test). **f** Induction of SULT1A1 expression in MCF7-WT cells treated with indicated concentrations of 4OH-TMX for 2 and 4 days, assessed by qRT-PCR (*p < 0.05, Student’s *t* test, comparison between 4OH-TMX versus DMSO treatment)

Several studies suggested a possible impact of SULT detoxification enzymes on the outcome of TMX therapy [8–11]. However, the data obtained so far were inconclusive. To find out whether SULT enzymes can contribute to TMX therapy resistance, we compared the expression of SULT family genes in patients with primary and relapsed metastatic breast cancers [30, 33, 34]. We found that the expression of the members of detoxification enzyme sulfotransferase family SULT1A1 and SULT1A2, but not other SULT family members (Additional file 1: Figure S1C), was induced in the metastatic breast cancers as compared to the primary tumors (Fig. 1a and Additional file 1: Figure S1A, B). In line with patient data, we detected about two- to three-fold increase of the level of SULT1A1 mRNA and protein in TMX-resistant LCC2 breast cancer cells, derivative of MCF7 cells, obtained by Brunner et al. [21] (Fig. 1b). Moreover, the CRISPR-Cas9-mediated deletion of *Sult1a1* alleles in spontaneous TMX^R^ MCF7 cells (Additional file 1: Figure S1D) resulted in more than 1.5–2-fold increase in sensitivity to 4OH-TMX (Fig. 1c and Additional file 1: Figure S1E). Taken together, these results point out to SULT1A1 as one of the factors conferring acquired resistance of patients to TMX therapy.

Next, we addressed the question whether the cells with increased SULT1A1 level are selected for by hormonal therapy. We treated freshly collected tumor cells from ER-positive breast cancer patients (Additional file 2: Table S1) and the ER-positive MCF7 cells with 4OH-TMX. Consistent with a previous study [35], we detected elevated SULT1A1 mRNA and protein levels (0.5-fold to 4-fold) in 4OH-TMX-treated patient-derived ER-positive breast cancer cells (Fig. 1d, e and Additional file 1: Figure S1F, G). Moreover, in MCF7 cells, 4 days treatment with 4OH-TMX leads to an increased (~ 2 fold) SULT1A1 mRNA level (Fig. 1f). Taken together, our data demonstrate that 4OH-TMX treatment results in the propagation of cancer cells with increased SULT1A1 expression. High SULT1A1 expression might serve as a potential biomarker for TMX resistance.

### Correlation between SULT1A1 expression and small molecules’ anticancer activity

After establishing a positive relationship between 4OH-TMX treatment and increased SULT1A1 expression in ex vivo and in vitro models, we reasoned that the combination of 4OH-TMX with anticancer compounds that require higher SULT1A1 levels for their anticancer activities might overcome the acquired TMX resistance. Therefore, we decided to find out whether there are anticancer compounds whose growth suppression activities depend on higher expression of SULT1A1. In order to search for such compounds, we analyzed the NCI-60 pharmacology database.

**Fig. 2:**
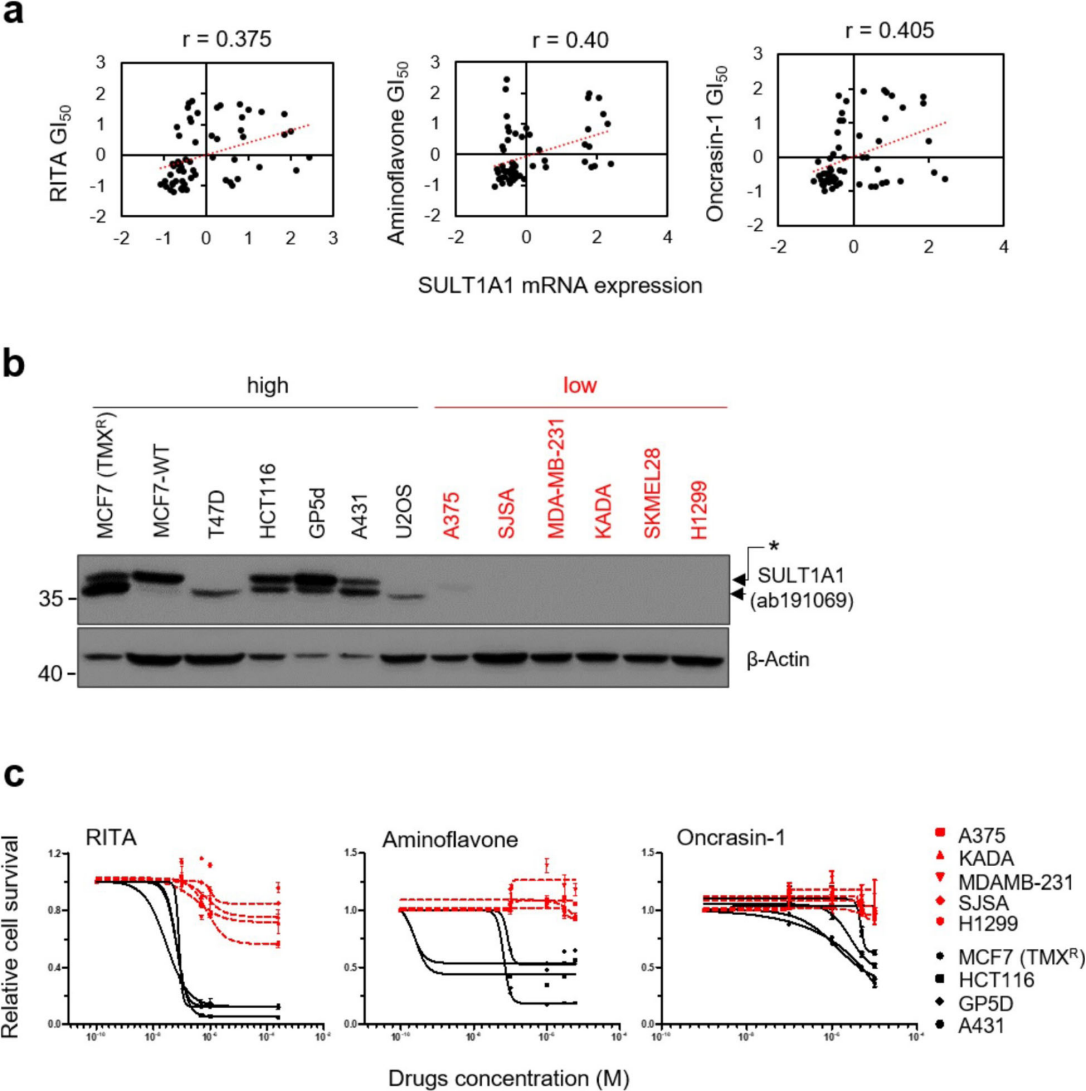
High SULT1A1 is the prerequisite for sensitivity to RITA, AF, and ONC-1. **a** Positive correlation of SULT1A1 mRNA level with sensitivity to RITA, AF, and ONC-1 in NCI-60 cell line database. Each black dot represents a cell line in NCI-60 database (“*r*” is the Pearson correlation coefficient). **b** SULT1A1 protein level in cancer cell lines. Breast cancer cells: MCF7, T47D, MDAMB-231; colon cancer: GP5d, HCT116; skin cancer: A431; osteosarcoma: U2OS, SJSA; melanoma: A375, KADA, SKMEL28; lung cancer: H1299. β-Actin was used as loading control. **c** RITA, AF, and Onc-1 efficiently suppress the growth of high SULT1A1-expressing cells (MCF7, HCT116, GP5d and A431, black solid line) while having negligible effect in cells with low SULT1A1 (A375, KADA, MDAMB-231, SJSA, H1299, red dotted line). Shown are the results obtained after 3 days of treatment, in three independent experiments

We discovered that the expression level of SULT1A1 positively correlates with cell toxicity upon treatment with small molecule RITA, Pearson correlation *r* = 0.375 (Fig. 2a, left panel). RITA was previously discovered by us in a screen for small molecules that selectively kill wild-type p53-expressing tumors, but not normal cells. RITA is highly cancer selective and showed antitumor activity in vitro and in vivo [12, 36]. The sensitivity to another two anticancer compounds, AF and ONC-1, also displayed a significant positive correlation with SULT1A1 expression, in line with previous reports [17, 18]. For AF Pearson correlation, *r* = 0.4, and for ONC-1, *r* = 0.405 (Fig. 2a).

To validate this correlation of anticancer activities by the compounds with SULT1A1 expression, we tested the effects of RITA, AF, and ONC-1 in nine cancer cell lines with different SULT1A1 levels (Fig. 2b). In line with the idea that SULT1A1 correlates with the sensitivity to RITA, AF, and ONC-1, four cell lines which express detectable SULT1A1 protein levels (in black) were highly sensitive to the compounds, while in those with nondetectable SULT1A1, these compounds had minimal or no effect (in red) (Fig. 2c and Additional file 1: Figure S2A, B). Collectively, these results suggest that the high SULT1A1 expression is either a biomarker for RITA, AF, and ONC-1 anticancer activity, or required for their cytotoxicity.

Thus, we set up experiments to find out if SULT1A1 plays a functional role and whether the antitumor efficacy of the compounds depends on SULT1A1.

### RITA, AF, and ONC-1 antitumor activity requires SULT1A1

**Fig. 3:**
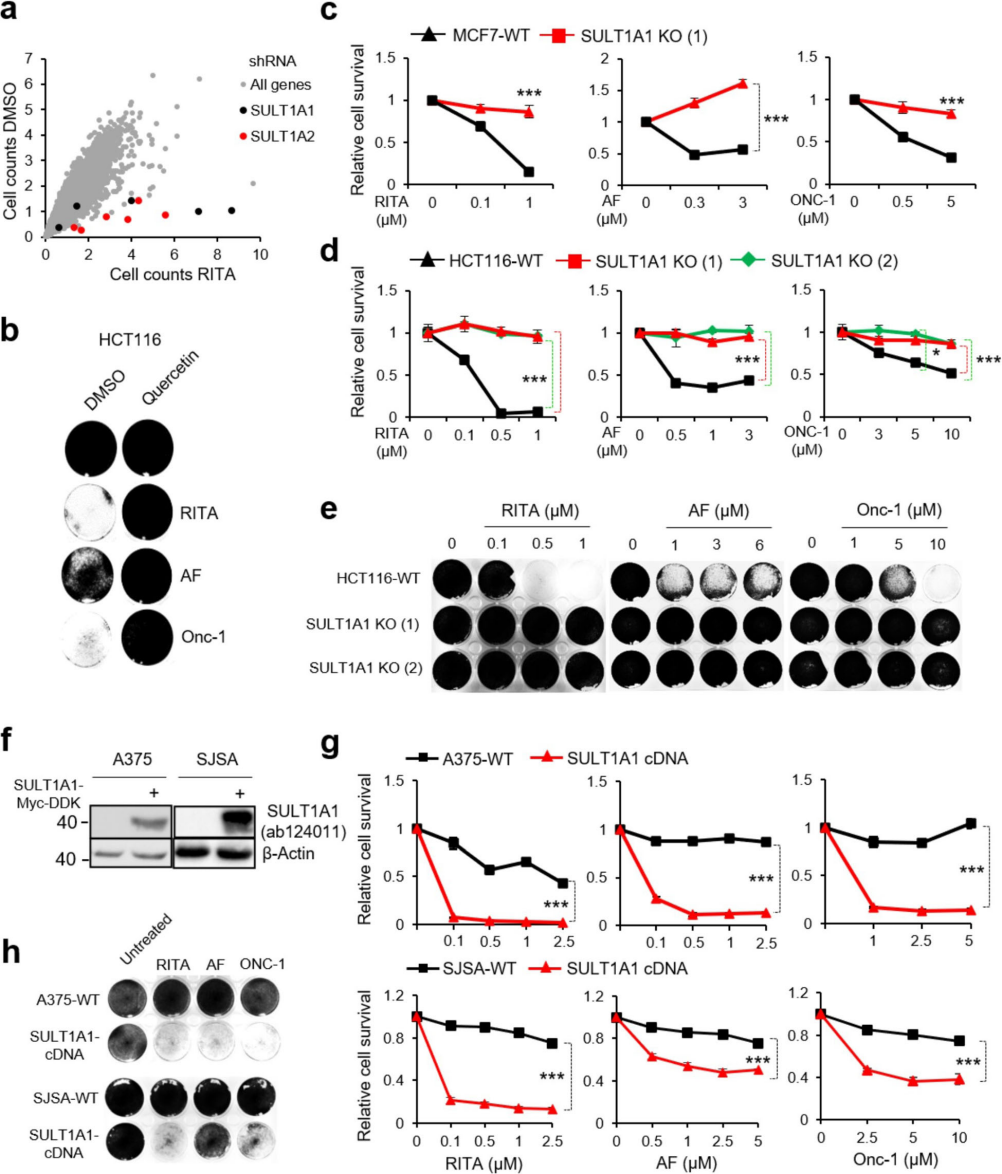
Antitumor activity of RITA, AF, and ONC-1 is SULT1A1-dependent. **a** The dot plot representation of genome-wide shRNA screen in MCF7 cells after DMSO and RITA treatment. Each dot represents a single shRNA. Black dots: shRNA against SULT1A1; red dots: shRNA against SULT1A2 genes; gray dots the rest of shRNAs. Each treatment was performed in triplicate and results for SULT1A1 and SULT1A2 had p 0.05. **b** Quercetin (10 μM) rescued growth suppression of HCT116 cells by RITA (1 μM), AF (3 μM), or ONC-1 (6 μM) as detected in the colony formation assay (72 h). Shown is crystal violet image. **c**, **d** Deletion of SULT1A1 conferred resistance to the three compounds. Shown is cell proliferation assay in SULT1A1-proficient parental cells MCF7 (**c**) and HCT116 (**d**), indicated in black line, and the SULT1A1 knockout clones #1 (red line) and #2 (green line), subjected to indicated concentrations of compounds. **e** RITA (left), AF (middle), and ONC-1 (right) suppressed the growth of HCT116 (WT) cells in a dose-dependent manner, but did not affect SULT1A1 KO clones, as assessed by a colony formation assay (as in **d**). **f** Overexpression of SULT1A1-Myc-DDK in A375 and SJSA cells detected using immunoblot. **g** SULT1A1 expression confers sensitivity to RITA, AF, and ONC-1 in low SULT1A1-expressing cells A375 (upper panel) and SJSA (lower panel). Control vector-transduced cells are indicated by a black line, and SULT1A1-transduced cells by a red line. **h** Sensitivity of SULT1A1-transduced cells to RITA, AF, and Onc-1 as assessed in colony formation assay. The values in **c**, **d**, and **g** represent mean ± SD of a triplicate (*p  0.05 and ***p  0.001, one-way ANOVA with Bonferroni’s multiple comparison test)

We previously performed a genome-wide shRNA knockdown screen using more than 27,000 shRNAs targeting about 5000 human genes in MCF7 cells to identify the gene set required for RITA-dependent apoptosis [29]. Indeed, we found that the members of SULTs family SULT1A1 and SULT1A2 are the top two genes in the screen: ablation of these genes rescued the cells from the effect of RITA (Fig. 3a).

To further validate the functional role of SULT1A1 expression for the antitumor activity of the compounds, we pretreated cells with plant-derived flavonoid Quercetin, known to inhibit the SULT enzyme activity, among other targets and activities [37]. Quercetin conferred resistance of SULT1A1-proficient HCT116 cells to all three compounds (Fig. 3b), strengthening the notion of a causal relationship between SULT1A1 activity and tumor suppression by RITA, AF, and ONC-1.

Further, we performed CRISPR/Cas9-mediated SULT1A1 knockout (KO) in HCT116 and MCF7 cells (Additional file 1: Figure S3A - D). *Sult1A1* deletion rescued growth suppression (Fig. 3c–e and Additional file 1: Figure S3E) upon treatment with the compounds in both KO lines, while the viability of control cells was suppressed by ~ 90–100%, 50–60%, and 40–60% by RITA, AF, and ONC-1, respectively. Finally, overexpression of wild-type SULT1A1 in low SULT1A1-expressing cells, i.e., A375 and SJSA (Fig. 3f), rendered them sensitive to the treatment with RITA, AF, and ONC-1 (Fig. 3g, h).

Overall, these data suggest that SULT1A1 is a prerequisite for the antitumor activity of the compounds.

### SULT1A1-dependent induction of oxidative stress via inhibition of TrxR1 activity is a major mechanism for antitumor activity of RITA, AF, and ONC-1

**Fig. 4:**
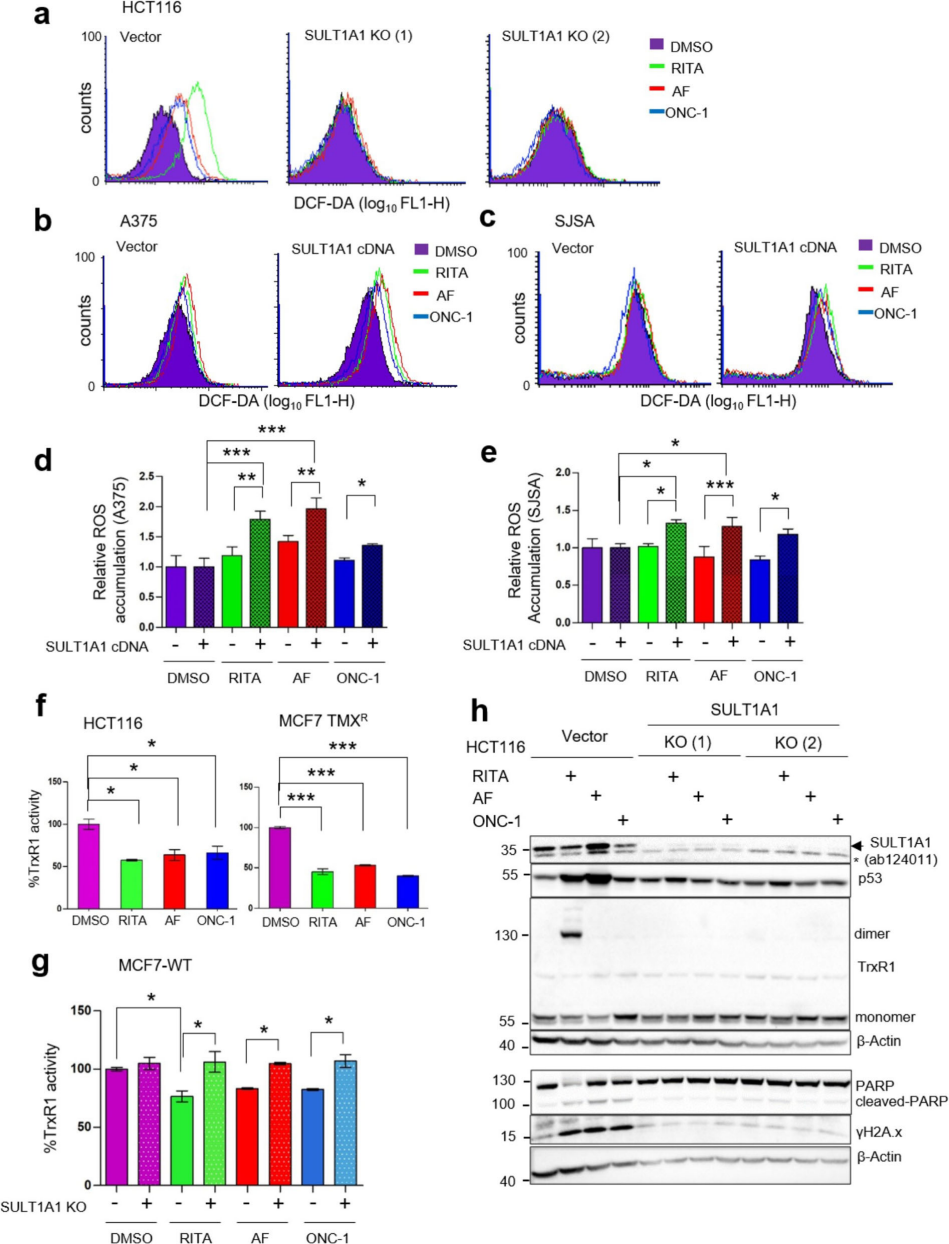
Inhibition of TrxR1 activity and induction of oxidative stress by RITA, AF, ONC-1 are SULT1A1-dependent. **a** SULT1A1-dependent induction of oxidative stress by 1 μM RITA (green), 3 μM AF (red), and 5 μM ONC-1 (blue) in HCT116 cells (24 h), but not in the derivative SULT1A1 KO lines. **b**, **c** Induction of oxidative stress in A375 (8 h) and SJSA (24 h) cells transfected with SULT1A1 cDNA, but not in vector-transfected cells after 1 μM RITA (green), 3 μM AF (red), and 1 μM ONC-1 (for A375) or 10 μM ONC-1 (for SJSA). Oxidative stress was measured using DCF-DA and followed by FACS analysis. **d**, **e** Histograms show the median ROS accumulation between compounds versus DMSO treatment, of n = 3 independent experiments in A375 (**d**) and SJSA (**e**) and *p  0.05 and ***p  0.001, one-way ANOVA with Bonferroni’s multiple comparison test. **f** Inhibition of TrxR1 activity in cell lysates after RITA (1 μM), AF (3 μM), and ONC-1 (5 μM) treatment for 6 h in HCT116 (left) and MCF7 TMX^R^ (right) as measured using TrxR-dependent Trx-coupled insulin disulfide reduction assay. Shown is percentage of activity compared to control (DMSO)-treated cells (mean ± SD n = 2 of independent experiments and *p  0.05 and ***p  0.001, one-way ANOVA with Bonferroni’s multiple comparison test). **g** SULT1A1-dependent inhibition of TrxR1 activity in MCF7, but not in isogenic SULT1A1 KO cell lysates, as described in **d**, except that the cells were treated for 10 h. Shown is percentage of activity compared to control DMSO-treated cells (mean ± SD n = 2, *p  0.05 and ***p  0.001, ANOVA with Bonferroni’s multiple comparison test). **h** SULT1A1-dependent induction of covalent TrxR1 dimer, apoptosis, and DNA damage in HCT116 cells and in its derivative SULT1A1 KO, treated or non-treated with RITA, AF, and ONC-1. Immunoblot of SULT1A1 (ab124011), p53, TrxR1, PARP, and γH2A.X (S139) is shown; β-actin was used as loading control

To get insight into the mechanism of the antitumor activity of SULT1A1-dependent compounds, we tested the level of oxidative stress upon treatment, using 2′-7′-dichlorodihydrofluorescein diacetate (DCFH-DA)-based assay. We found a significant increase in DCF fluorescence only in SULT1A1-proficient cells, i.e., HCT116 with high SULT1A1 level, but not in the SULT1A1 KO derivatives (Fig. 4a). In line with these results, SULT1A1 dependence was observed in A375 and SJSA cells transfected with SULT1A1-Myc-DDK-tagged cDNA, but not in the empty vector-transfected cells (Fig. 4b–e). Oxidative stress induction can sometimes be rescued by the redox-active and often studied compound resveratrol. Co-treatment of RITA, AF, and ONC-1 with resveratrol indeed lowered the DCF fluorescence by 2.8-, 2-, or 1.42-fold, respectively (Additional file 1: Figure S4A).

Cancer cells often display an elevated oxidative stress burden and therefore are more susceptible to further increased level of reactive oxygen species (ROS) [38]. One of the promising targets for anticancer therapy is the antioxidant defense enzyme NADPH-dependent selenoprotein thioredoxin reductase (TrxR), often overexpressed in cancer [39]. Recent studies demonstrate that cancers are highly dependent on the antioxidant and redox regulating thioredoxin (Trx) and glutaredoxin systems [40]. Therefore, targeting cancer vulnerability by inhibiting either the thioredoxin or glutaredoxin system could pave a way to more cancer-specific therapies. We addressed the question whether the three compounds can target these antioxidant systems in cells. We found that all three compounds inhibit the activity of TrxR1, as tested in colon cancer cells HCT116 and in breast cancer MCF7 TMX^R^ and WT cells (Fig. 4f, g). Notably, the depletion of SULT1A1 prevented the inhibitory effect of the compounds on TrxR1 (Fig. 4g), which was associated with the loss of elevated oxidative stress (Fig. 4a) and decreased apoptosis, as evidenced by PARP cleavage (Fig. 4h). These results provide a strong evidence that SULT1A1 activity is required for the inhibition of TrxR1, induction of oxidative stress and triggering apoptosis by RITA, AF, and ONC-1.

In agreement with our previous reports [32, 41], RITA inhibited TrxR1 concomitantly with an induction of covalently linked TrxR1 dimers of ~ 130 kDa molecular weight (Fig. 4h). Importantly, we did not detect such TrxR1 covalently linked dimer formation upon RITA treatment using SULT1A1-deficient isogenic cells (Fig. 4h and Additional file 1: Figure S4B), supporting the notion that SULT1A1 is crucial for the inhibition of TrxR1 by RITA. Further evidence for the importance of SULT1A1 for the inhibition of TrxR1 by RITA comes from the overexpression of SULT1A1 in cells with low SULT1A1 levels, A375 and SJSA, which resulted in the formation of the TrxR1 covalent dimer upon RITA treatment (Additional file 1: Figure S4C, D). We did not observe that TrxR1 covalent dimer formation after AF and ONC-1 treatment, suggesting that AF and ONC-1 might inhibit the TrxR1 activity without crosslinking of the Trp114 residues of TrxR1 which we found to yield the covalent dimer formation triggered by RITA [32, 41].

Collectively, our findings suggest a common mechanism of oxidative stress induction by the compounds via SULT1A1-dependent inhibition of TrxR1 activity.

Additionally, we reasoned that increased oxidative stress might induce DNA damage signaling. Indeed, the level of well-known DNA damage marker, phosphorylated S139 H2AX (γH2AX), was upregulated only in SULT1A1-proficient cells upon treatments and was followed by p53 induction (Fig. 4h and Additional file 1: Figure S4C - D). Resveratrol mitigated the γH2AX level, induction of p53 and apoptosis marker, cleaved PARP (Additional file 1: Figure S4E), which was in line with our previous study [20]. These data demonstrated that the induction of DNA damage signaling, and apoptosis is likely to be dependent on the increased oxidative stress. Since the compound-induced γH2AX and apoptosis were absent in cells with low SULT1A1 level but were readily induced by overexpression of SULT1A1 (Additional file 1: Figure S4C, D), we conclude that these effects of compounds are mediated by SULT1A1.

Taken together, our results demonstrated that the induction of apoptosis by RITA, AF, and ONC-1 is due to the SULT1A1-dependent inhibition of TrxR1 activity, followed by the induction of oxidative stress and DNA damage signaling.

### Pretreatment with 4OH-TMX confers vulnerability of breast cancer patient-derived cells to SULT1A1-dependent anticancer compounds

**Fig. 5:**
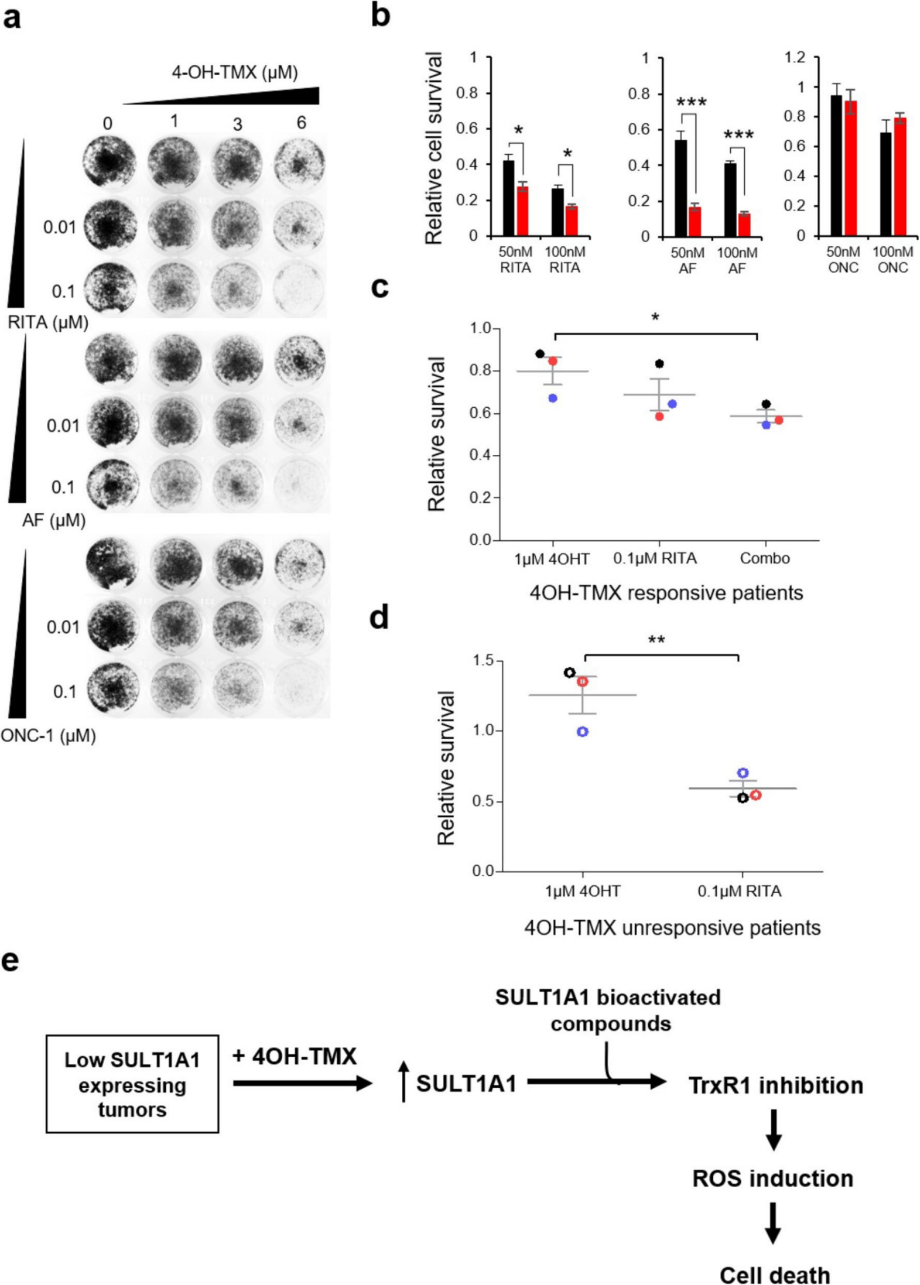
Tamoxifen sensitizes ER-positive breast cancer to RITA. **a** Synergy between 4OH-TMX and low dose of compounds in estrogen-positive MCF7 cells. Long-term viability assay (12 days) is shown. **b** TMX-resistant LCC2 cells (red) are more sensitive to RITA and AF, than MCF7 (WT) cells. Shown is the difference in growth suppression between cell lines treated for 72 h with indicated concentration of compounds (mean ± SD *n* = 6, *p < 0.05 and ***p < 0.001, one-way ANOVA with Bonferroni’s multiple comparison test). **c** Pretreatment of 4-OH-TMX (1 μM, 6 days) sensitize the ex vivo cultured breast cancer patient cells from patients #K5, (blue solid dots); #K6 (red dots), and #K10 (black dots) to low dose of RITA (100 nM, treatment for 3 days post-4OH-TMX). Shown is mean ± SD, *p < 0.05, one-way ANOVA with Bonferroni’s multiple comparison test. **d** Breast cancer patient cells from patients #K7 (black dots), #K8 (blue dots), and #K9 (red dots) that were unresponsive to 4OH-TMX treatment show sensitivity to 100 nM RITA (mean ± SD, **p < 0.01, Student’s *t* test). **e** Model illustrating how SULT1A1-dependent compounds can help to overcome TMX resistance. 4OH-TMX-treated relapsed breast tumors display upregulation of SULT1A1, leading to drug resistance. This creates vulnerability to treatment with SULT1A1-dependent RITA, AF, and ONC-1, which eliminate cancer cells by inhibition of TrxR1 and induction of oxidative stress, DNA damage, and apoptosis

Considering our findings that SULT1A1 expression is induced by 4OH-TMX treatment (Fig. 1), and that SULT1A1 expression is required for RITA, AF, ONC-1 antitumor activity, we hypothesized that the compounds would kill TMX-pretreated cancer cells more efficiently. Indeed, we observed synergy upon the combination of 4OH-TMX pretreatment with a low dose of compounds, as tested in MCF7-WT cells (Fig. 5a). Comparison of the response of TMX-sensitive MCF7 (WT) and TMX-resistant TMX^R^ LCC2 cells revealed a significantly increased sensitivity to RITA (~ 1.5-fold–2-fold) and AF (~ 3-fold) of TMX^R^ cells (Fig. 5b). However, there was no significant difference in sensitivity to ONC-1 in the WT and TMX^R^ cells, suggesting additional mechanisms for ONC-1, and/or additional mutations in LCC2 cells (Fig. 5b).

Having established the therapeutic potential of RITA and AF for 4OH-TMX-pretreated cells in vitro, we further administrated the same treatments ex vivo on tumor cell cultures from ER-positive breast cancer patients (Additional file 2: Table S1). Notably, we observed an additive effect of 4OH-TMX and RITA combination in TMX-sensitive breast cancer samples (Fig. 5c). Moreover, low dose of RITA achieved a significant growth reduction in patient-derived cancer cells that were unresponsive to TMX treatment (Fig. 5d). Taken together, these results provide the evidence supporting the hypothesis of utilizing SULT1A1-activated compounds to overcome TMX resistance for recurrent ER-positive breast cancer patients (Fig. 5e).

## Discussion

Use of tamoxifen as the principal therapy for postmenopausal women diagnosed with estrogen receptor (ER)-positive breast cancer has contributed significantly to the survival of patients. However, around 30% of women experience relapse within the first 5 years of tamoxifen therapy [4, 5]. Therefore, novel combination therapies as well as biomarkers are needed to help patients. Since monotherapy cannot cure cancer, it is imperative to find those drug combinations which would help in fighting TMX resistance and cancer recurrence. Here, we identified a potential biomarker of TMX resistance, SULT1A1, which is upregulated after TMX treatment in metastatic breast cancers in relapsed patients, in treated breast cancer samples ex vivo and in breast cancer cell lines in vitro. Moreover, we found that increased SULT1A1 level renders selective vulnerability to three anticancer compounds, whose mechanism of action we found to be SULT1A1-dependent. We validated this finding in breast cancer patient samples ex vivo. Thus, we identified a novel therapeutic combination, which could be used in clinic to overcome the problem of TMX resistance and improve the survival of patients.

SULT1A1 is a phase II enzyme, a member of the sulfotransferase family, which has the capability to sulfate xenobiotics and endogenous chemicals as a metabolic step towards neutralizing them [42]. Sulfotransferase 1A1 performs the detoxification of 4-OH-TMX [8]. Our finding of the association of increased resistance to tamoxifen with high level of SULT1A1 and that TMX-resistant cells are rendered sensitive to 4OH-TMX treatment after SULT1A1 KO is in agreement with the notion that detoxification of 4-OH-TMX by SULT1A1 is neutralizing its activity.

Previous clinical studies addressed the role of polymorphism in SULT1A1 in the survival of breast cancer patients. However, these studies have produced conflicting results, which indicate either a better survival upon TMX therapy of breast cancer patients carrying SULT1A1 allele encoding enzyme with low activity [9] or, in contrast, worse survival of such patients [10, 43], or no impact whatsoever [35]. It should be noted that the outcome of TMX therapy is not solely determined by a single detoxification enzyme but a combination of several factors, including by uridine diphosphate glucuronosyltransferases (UGTs), polymorphisms associated with the CYP genes, especially the CYP2D6, and others [44]. This complexity of TMX metabolism may explain the discrepancies observed in different clinical studies mentioned above. However, there has been no clinical study so far addressing the role of the level of SULT1A1 expression in relation to patient survival. More systematic clinical studies, including more patients, and considering several aspects related to TMX metabolizing enzymes, will shed light on this issue.

While we found an increased level of SULT1A1 after treatment with tamoxifen, the exact mechanism of this phenomenon is unclear. 4-OH-TMX might mediate the induction of SULT1A1 expression on mRNA level, as it has been shown earlier [35]. It is possible that TMX enhances the recruitment of the transcription factor SP1, required for the activation of SULT1A1 expression [45], to its promoter, as it has been shown for genes encoding p21 and p27 [46, 47]. Since we demonstrate here that high expression of SULT1A1 confer resistance to 4OH-TMX, it is also possible that the elevated expression of SULT1A1 in cell population after treatment with 4OH-TMX was due to the preferable survival of cells with intrinsic high SULT1A1 level.

Interestingly, SULT1A1 has also been shown to bio-transform pro-drugs into their active form [17–19, 48]. Our experiments, using a number of approaches and cell models, including genome-wide shRNA screen, CRISPR/Cas9-mediated SULT1A1 deletion versus its overexpression, and analysis of more than a dozen of cancer cell lines, provide a strong evidence of SULT1A1 dependence of growth suppression by three anticancer compounds, RITA, AF, and ONC-1. While this phenomenon was indicated in previous studies [17–19], the reason for it was not clear.

RITA, AF, and ONC-1 cause tumor regression in several cancer cell models and mouse tumor models without noticeable adverse effects, making them promising candidates for anticancer treatment [13, 49–52]. The mechanism of action of these compounds was addressed in several studies, including ours, but their most relevant target(s) remain a subject of debate [12, 17, 18]. Tumor suppression by RITA, AF, and ONC-1 has been linked to the induction of ROS [15, 32, 41, 53, 54]. Importantly, in this study, we identified a common target for all three compounds, which is affected by them in a SULT1A1-dependent manner. We found that RITA, AF, and ONC-1 inhibit TrxR1 and induce oxidative stress in cancer cells in a SULT1A1-dependent manner.

Increased oxidative stress, associated with enhanced proliferation and altered metabolism, is one of the vulnerabilities of cancer cells which could be exploited by targeting ROS-neutralizing enzymes. NADPH-dependent selenoprotein thioredoxin reductase (TrxR) is often overexpressed in cancer [39, 55], which associates with poor survival of patients with different types of cancer, including breast, lung, pancreatic, prostate, and head and neck cancers [39]. This makes TrxR1 a promising target for the development of anticancer drugs, especially considering that in part due to the selenoprotein nature of this enzyme it is particularly prone to inhibition by electrophilic compounds [40]. From the therapeutic perspective, it is interesting to note an opposite effect of TrxR1 inhibition on normal and cancer cells: survival of normal cells or even strengthening them against oxidative stress versus death of cancer cells [39]. In our recent study, we have demonstrated that the three compounds induce exaggerated oxidative stress selectively in cancer cells, which is a prerequisite for targeting the mRNA transcription machinery. Thus, these compounds attack cancer vulnerability—transcriptional addiction. Inhibition of transcription machinery results in preferential inhibition of major oncogenic pathways, thus killing cancer cells [20].

In summary, the high level of SULT1A1, while conferring resistance to TMX, provides a selective vulnerability to SULT1A1-dependent compounds. Our ex vivo experiments using breast cancer samples demonstrated the efficiency of the combination of TMX with SULT1A1-dependent compounds, both in TMX-sensitive and TMX-resistant patients. Thus, our study provides a strong mechanistic support for novel combinatorial treatment of relapsed patients with SULT1A1-activated compounds, such as RITA and AF. When patients recur on TMX, they are usually treated with aromatase inhibitors (AI), often the main alternative for high-risk patients. However, premenopausal women cannot be treated with AI since the endogenous estrogen production is too high. We speculate that combining TMX with SULT1A1-dependent compounds could provide a therapeutic option for young patients. Our results might pave a way to a strategy to circumvent TMX resistance and suggest that this approach could be developed further for the benefit of patients.

## Conclusions

We provide an ample evidence that a high level of the detoxifying enzyme SULT1A1 confers resistance to tamoxifen therapy in both in vitro and ex vivo models and correlates with TMX resistance in metastatic samples in relapsed patients. Moreover, high SULT1A1 provides selective vulnerability to anticancer compounds RITA, AF, and ONC-1 and lay out a rationale for a new combination therapy to overcome TMX resistance in young breast cancer patients.

## Supplementary information

**Additional file 1: Figure S1.** Increased SULT1A1 mRNA and protein expression in relapsed patients after TMX treatment. A, B, Heat map representation of microarray analysis of matched primary and metastatic tumors from patients A – F (Table S2). The levels of SULT1A1 (A) and SULT1A2 (B) expression is compared with primary tumor after TMX treatment. C, Heatmap of SULTs family genes differentially expressed in matched primary tumor and liver metastasis samples from breast cancer patients as assessed by RNA-seq. D, Representative WB for SULT1A1 KO clones in spontaneous TMX^R^ MCF7 cells. β-actin used as loading control. The asterisk indicates non-specific band produced by antibody. E, SULT1A1 deletion (blue line, #10) in spontaneous TMX^R^ clone of MCF7 cells (red line) confers sensitivity after 4 days post treatment with different concentrations of 4OH-TMX, as determined by resazurin assay (***p < 0.001, one-way ANOVA with Bonferroni’s multiple comparison test). F, Representative immunofluorescence images showing increased SULT1A1 protein (in red, using ab124011 antibody) after treatment of breast cancer patient #K4 ex vivo cultured cells with either vehicle or 1 μM of 4OH-TMX for 6 days. G, qRT-PCR of SULT1A1 mRNA in patient samples treated with TMX as in F. Patient information is given in Table S1. **Figure S2.** SULT1A1 is required for RITA, AF and ONC-1 sensitivity in cancer cells. A, Crystal violet staining detecting cell viability of high (MCF7 and T-47D) and low SULT1A1 (A375 and SJSA) after 72 h treatment with the indicated concentrations of compounds. B, SULT1A1 protein expression in cancer cell lines. Breast cancer cells: MCF7 TMX^R^, MDAMB-231; colon cancer: GP5d, HCT116; lung cancer: H1299; melanoma: A375, KADA, SKMEL28, SKMEL2, ESTDAB-37; neuroblastoma: SHSY-5Y; osteosarcoma: U2OS; skin cancer: A431. β-actin used as loading control. SULT1A1 (ab124011) antibody was used to perform the WB. **Figure S3.** Generation and validation of SULT1A1 KO in cancer cells. A - D, Generation of SULT1A1 knock-out clones in MCF7 and HCT116 cells using CRISPR/Cas9 gene editing. A, C and D, shown is immunoblot images using SULT1A1 (ab191069) antibody in MCF7-WT (A), HCT116 (D) and in HCT116 using SULT1A1 (ab124011) antibody (C). β-actin used as loading control. The asterisk indicates non-specific band produced by the antibody ab191069 and ab124011 and B, Immunofluorescence analysis of SULT1A1 expression in MCF7-vector transduced and SULT1A1 KO clone using ab124011 antibody (in red). E, Crystal violet staining to detect cell viability in long-term viability assay on day 6 after compounds treatment of MCF7-WT and SULT1A1 KO clones 1 and 3. **Figure S4.** Inhibition of TrxR1 activity by RITA, AF, and ONC-1 is SULT1A1 dependent. A, Increased oxidative stress upon 24 h treatment by 1 μM RITA (green), 3 μM AF (red) and 5 μM ONC-1 (blue) in HCT116 cells was rescued by co-treatment with 1 μM resveratrol (filled pattern). B, Immunoblot of TrxR1 and β-actin as loading control in the lysates from MCF7-WT cells. C, D, Induction of covalent TrxR1 dimer (detected by TrxR1 Antibody), apoptosis (induction of cleaved PARP) and DNA damage (yH2A.X (p-S139)) in SULT1A1-overexpressing cells. Shown Immunoblot of SULT1A1, p53, TrxR1, PARP and ϒH2A.X in cell extracts from low SULT1A1-expressing A375 (C), SJSA (D) compared to SULT1A1 overexpressing cells. β-Actin was used as loading control. E, Induction of γH2A.X (S139) and PARP, indicators of DNA damage and apoptosis, respectively, was rescued by the treatment with redox active compound resveratrol (1 μM), as assessed by immunoblot.

**Additional file 2:Table S1.** Patient’s information for survival, Immunofluorescence, and qPCR. **Table S2.** Patient’s information for microarray analysis.

## Data Availability

Not applicable.
