# Supplementary figures and images for: Identification and targeting of selective vulnerability rendered by tamoxifen resistance
Source: Breast Cancer Res. 2020 Jul 29;22:80. doi: 10.1186/s13058-020-01315-5 (PMC7388523; doi:10.1186/s13058-020-01315-5)

Figure S1.

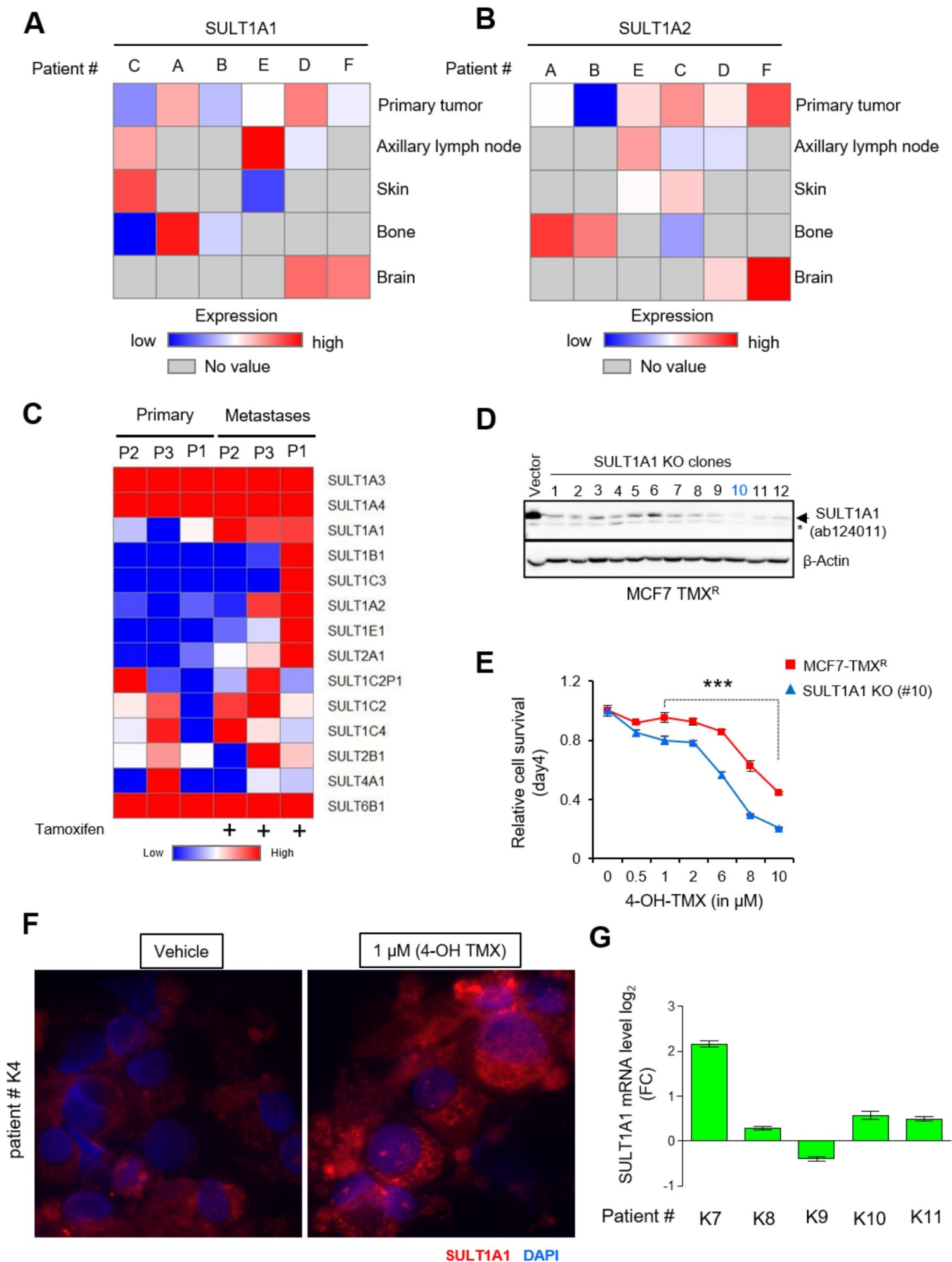

Supplement: Supplementary file 1 — Additional file 1: Figure S1. Increased SULT1A1 mRNA and protein expression in relapsed patients after TMX treatment. A, B, Heat map representation of microarray analysis of matched primary and metastatic tumors from patients A – F (Table S2). The levels of SULT1A1 (A) and SULT1A2 (B) expression is compared with primary tumor after TMX treatment. C, Heatmap of SULTs family genes differentially expressed in matched primary tumor and liver metastasis samples from breast cancer patients as assessed by RNA-seq. D, Representative WB for SULT1A1 KO clones in spontaneous TMXR MCF7 cells. β-actin used as loading control. The asterisk indicates non-specific band produced by antibody. E, SULT1A1 deletion (blue line, #10) in spontaneous TMXR clone of MCF7 cells (red line) confers sensitivity after 4 days post treatment with different concentrations of 4OH-TMX, as determined by resazurin assay (***p < 0.001, one-way ANOVA with Bonferroni’s multiple comparison test). F, Representative immunofluorescence images showing increased SULT1A1 protein (in red, using ab124011 antibody) after treatment of breast cancer patient #K4 ex vivo cultured cells with either vehicle or 1 μM of 4OH-TMX for 6 days. G, qRT-PCR of SULT1A1 mRNA in patient samples treated with TMX as in F. Patient information is given in Table S1. Figure S2. SULT1A1 is required for RITA, AF and ONC-1 sensitivity in cancer cells. A, Crystal violet staining detecting cell viability of high (MCF7 and T-47D) and low SULT1A1 (A375 and SJSA) after 72 h treatment with the indicated concentrations of compounds. B, SULT1A1 protein expression in cancer cell lines. Breast cancer cells: MCF7 TMXR, MDAMB-231; colon cancer: GP5d, HCT116; lung cancer: H1299; melanoma: A375, KADA, SKMEL28, SKMEL2, ESTDAB-37; neuroblastoma: SHSY-5Y; osteosarcoma: U2OS; skin cancer: A431. β-actin used as loading control. SULT1A1 (ab124011) antibody was used to perform the WB. Figure S3. Generation and validation of SULT1A1 KO in cancer cells. A - D, Gener [file 13058_2020_1315_MOESM1_ESM.zip › FFigure S1.pdf]

Figure S2.

A

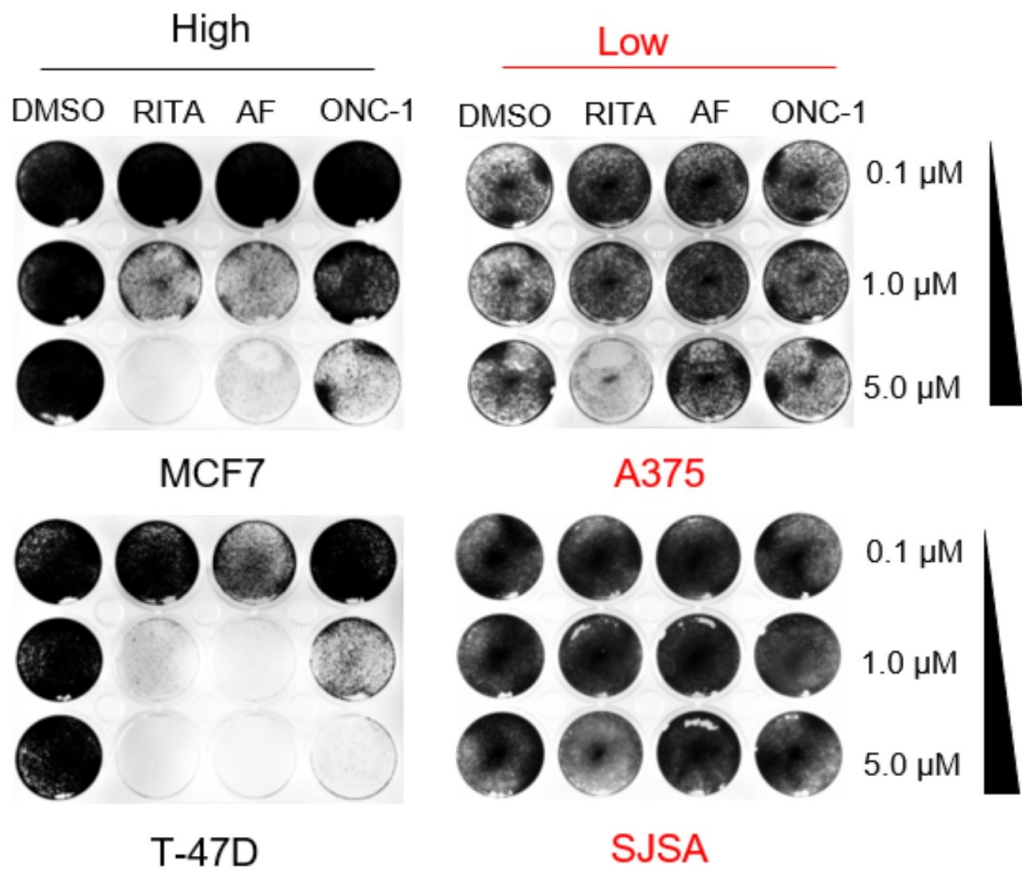

B

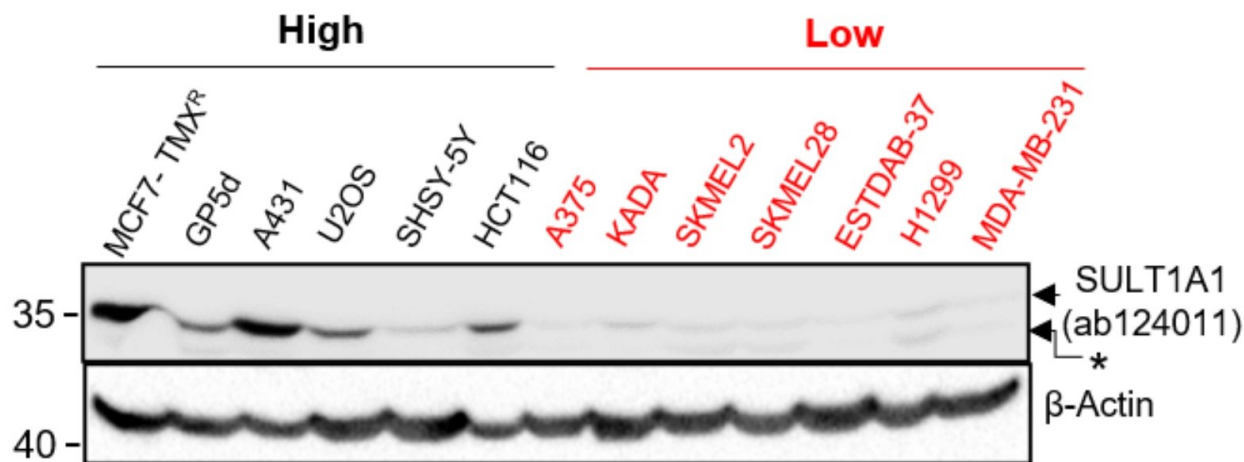

Supplement: Supplementary file 1 — Additional file 1: Figure S1. Increased SULT1A1 mRNA and protein expression in relapsed patients after TMX treatment. A, B, Heat map representation of microarray analysis of matched primary and metastatic tumors from patients A – F (Table S2). The levels of SULT1A1 (A) and SULT1A2 (B) expression is compared with primary tumor after TMX treatment. C, Heatmap of SULTs family genes differentially expressed in matched primary tumor and liver metastasis samples from breast cancer patients as assessed by RNA-seq. D, Representative WB for SULT1A1 KO clones in spontaneous TMXR MCF7 cells. β-actin used as loading control. The asterisk indicates non-specific band produced by antibody. E, SULT1A1 deletion (blue line, #10) in spontaneous TMXR clone of MCF7 cells (red line) confers sensitivity after 4 days post treatment with different concentrations of 4OH-TMX, as determined by resazurin assay (***p < 0.001, one-way ANOVA with Bonferroni’s multiple comparison test). F, Representative immunofluorescence images showing increased SULT1A1 protein (in red, using ab124011 antibody) after treatment of breast cancer patient #K4 ex vivo cultured cells with either vehicle or 1 μM of 4OH-TMX for 6 days. G, qRT-PCR of SULT1A1 mRNA in patient samples treated with TMX as in F. Patient information is given in Table S1. Figure S2. SULT1A1 is required for RITA, AF and ONC-1 sensitivity in cancer cells. A, Crystal violet staining detecting cell viability of high (MCF7 and T-47D) and low SULT1A1 (A375 and SJSA) after 72 h treatment with the indicated concentrations of compounds. B, SULT1A1 protein expression in cancer cell lines. Breast cancer cells: MCF7 TMXR, MDAMB-231; colon cancer: GP5d, HCT116; lung cancer: H1299; melanoma: A375, KADA, SKMEL28, SKMEL2, ESTDAB-37; neuroblastoma: SHSY-5Y; osteosarcoma: U2OS; skin cancer: A431. β-actin used as loading control. SULT1A1 (ab124011) antibody was used to perform the WB. Figure S3. Generation and validation of SULT1A1 KO in cancer cells. A - D, Gener [file 13058_2020_1315_MOESM1_ESM.zip › Figure S2.pdf]

Figure S3.

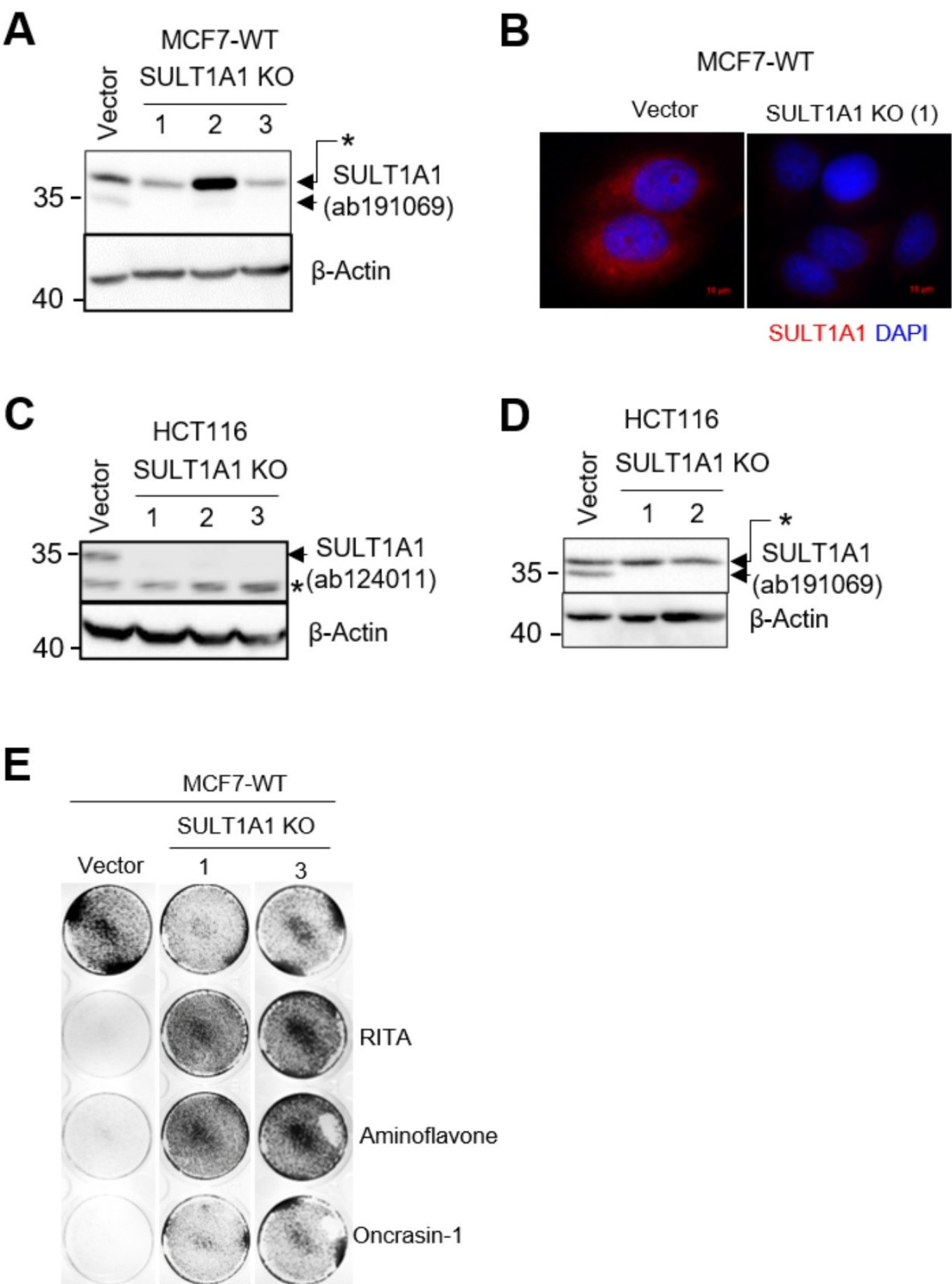

Supplement: Supplementary file 1 — Additional file 1: Figure S1. Increased SULT1A1 mRNA and protein expression in relapsed patients after TMX treatment. A, B, Heat map representation of microarray analysis of matched primary and metastatic tumors from patients A – F (Table S2). The levels of SULT1A1 (A) and SULT1A2 (B) expression is compared with primary tumor after TMX treatment. C, Heatmap of SULTs family genes differentially expressed in matched primary tumor and liver metastasis samples from breast cancer patients as assessed by RNA-seq. D, Representative WB for SULT1A1 KO clones in spontaneous TMXR MCF7 cells. β-actin used as loading control. The asterisk indicates non-specific band produced by antibody. E, SULT1A1 deletion (blue line, #10) in spontaneous TMXR clone of MCF7 cells (red line) confers sensitivity after 4 days post treatment with different concentrations of 4OH-TMX, as determined by resazurin assay (***p < 0.001, one-way ANOVA with Bonferroni’s multiple comparison test). F, Representative immunofluorescence images showing increased SULT1A1 protein (in red, using ab124011 antibody) after treatment of breast cancer patient #K4 ex vivo cultured cells with either vehicle or 1 μM of 4OH-TMX for 6 days. G, qRT-PCR of SULT1A1 mRNA in patient samples treated with TMX as in F. Patient information is given in Table S1. Figure S2. SULT1A1 is required for RITA, AF and ONC-1 sensitivity in cancer cells. A, Crystal violet staining detecting cell viability of high (MCF7 and T-47D) and low SULT1A1 (A375 and SJSA) after 72 h treatment with the indicated concentrations of compounds. B, SULT1A1 protein expression in cancer cell lines. Breast cancer cells: MCF7 TMXR, MDAMB-231; colon cancer: GP5d, HCT116; lung cancer: H1299; melanoma: A375, KADA, SKMEL28, SKMEL2, ESTDAB-37; neuroblastoma: SHSY-5Y; osteosarcoma: U2OS; skin cancer: A431. β-actin used as loading control. SULT1A1 (ab124011) antibody was used to perform the WB. Figure S3. Generation and validation of SULT1A1 KO in cancer cells. A - D, Gener [file 13058_2020_1315_MOESM1_ESM.zip › Figure S3.pdf]

Figure S4.

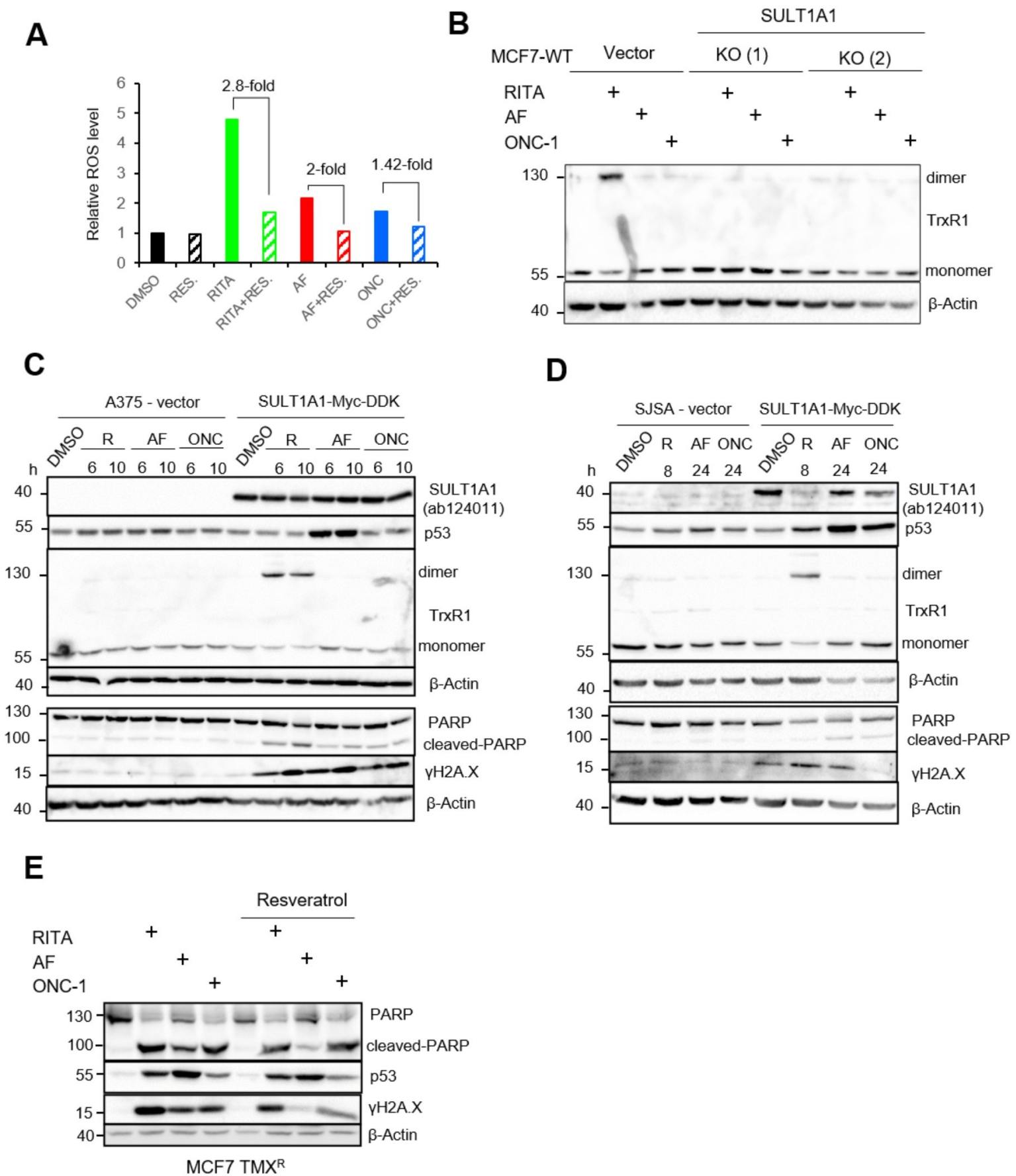

Supplement: Supplementary file 1 — Additional file 1: Figure S1. Increased SULT1A1 mRNA and protein expression in relapsed patients after TMX treatment. A, B, Heat map representation of microarray analysis of matched primary and metastatic tumors from patients A – F (Table S2). The levels of SULT1A1 (A) and SULT1A2 (B) expression is compared with primary tumor after TMX treatment. C, Heatmap of SULTs family genes differentially expressed in matched primary tumor and liver metastasis samples from breast cancer patients as assessed by RNA-seq. D, Representative WB for SULT1A1 KO clones in spontaneous TMXR MCF7 cells. β-actin used as loading control. The asterisk indicates non-specific band produced by antibody. E, SULT1A1 deletion (blue line, #10) in spontaneous TMXR clone of MCF7 cells (red line) confers sensitivity after 4 days post treatment with different concentrations of 4OH-TMX, as determined by resazurin assay (***p < 0.001, one-way ANOVA with Bonferroni’s multiple comparison test). F, Representative immunofluorescence images showing increased SULT1A1 protein (in red, using ab124011 antibody) after treatment of breast cancer patient #K4 ex vivo cultured cells with either vehicle or 1 μM of 4OH-TMX for 6 days. G, qRT-PCR of SULT1A1 mRNA in patient samples treated with TMX as in F. Patient information is given in Table S1. Figure S2. SULT1A1 is required for RITA, AF and ONC-1 sensitivity in cancer cells. A, Crystal violet staining detecting cell viability of high (MCF7 and T-47D) and low SULT1A1 (A375 and SJSA) after 72 h treatment with the indicated concentrations of compounds. B, SULT1A1 protein expression in cancer cell lines. Breast cancer cells: MCF7 TMXR, MDAMB-231; colon cancer: GP5d, HCT116; lung cancer: H1299; melanoma: A375, KADA, SKMEL28, SKMEL2, ESTDAB-37; neuroblastoma: SHSY-5Y; osteosarcoma: U2OS; skin cancer: A431. β-actin used as loading control. SULT1A1 (ab124011) antibody was used to perform the WB. Figure S3. Generation and validation of SULT1A1 KO in cancer cells. A - D, Gener [file 13058_2020_1315_MOESM1_ESM.zip › Figure S4.pdf]
